# Stress Processes Over 2 Decades: Findings From the National Study of Daily Experiences

**DOI:** 10.1093/geroni/igab046.822

**Published:** 2021-12-17

**Authors:** David Almeida, Daniel Mroczek

## Abstract

Daily experiences of stress and the associated sequelae of affective and physiological changes represent the multiple dimensions of a complex, time-dependent process of how stressors unfold in daily life. Daily diaries capture these time-sensitive processes as they occur under real world conditions. Longitudinal changes in stress processes can then be tracked using a measurement burst design: daily diaries repeated longitudinally. Using this design, the National Study of Daily Experiences (NSDE) has generated more than 35,000 days of data from a national sample of over 2,500 adults assessed repeatedly across 20 years of adulthood. The NSDE features details of more than 10,000 reports daily stress including exposure, appraisal and affective responses from adults ranging in age from 24 to 95 years. The current symposium leverages this unique and influential dataset to examine age differences and aging-related changes in daily stress processes with four presentations from the NSDE. First, Dr. Robert Stawski will discuss longitudinal change and age-related differences in exposure to multiple types of daily stressors. Next, Dr. Susan Charles will examine age differences and change in a key element of the stress process: negative affect. Third, Dr. Eric Cerino will describe longitudinal change in appraisals of daily stressors focusing on stressor control. Finally, Dr. David Almeida will examine changes in negative affect reactivity to daily stressors across the 20 years of the NSDE. Dr. Dan Mroczek will discuss the picture these presentations provide of how aging and age-differences impact the daily stress process and future directions for understanding these trajectories.

